# GC–MS‐Based Metabolomic Insights Into Fermentation‐Induced Bioactive Compounds and Metabolic Health‐Related Activities of White Corn Steep Liquor

**DOI:** 10.1002/fsn3.71884

**Published:** 2026-05-27

**Authors:** Lemohang Gumenku, Ochuko L. Erukainure, S'thandiwe N. Magwaza, Almahi I. Mohamed, Md. Shahidul Islam, Ademola O. Olaniran

**Affiliations:** ^1^ Department of Microbiology, School of Life Sciences University of KwaZulu‐Natal Durban South Africa; ^2^ Department of Biochemistry, School of Life Sciences University of KwaZulu‐Natal Durban South Africa

**Keywords:** biochemical dynamics, metabolic disease, steep liquor, white corn

## Abstract

The present study investigated the effect of crude fermentation on white corn steep liquor and its bioactive constituents in the management of metabolic diseases. White corn steep liquor was prepared and fermented for 24, 48, and 72 h. The liquors were profiled with GC–MS, and the identified metabolites were subjected to metabolomics analyses using MetaboAnalyst 6.0. The antioxidant activities of the liquors were evaluated using 2,2‐diphenyl‐1‐picrylhydrazyl (DPPH) and Ferric Reducing Antioxidant Power (FRAP) assays. The antidiabetic activities of the liquors were evaluated via their inhibitory activities on α‐amylase and α‐glucosidase, while their antiobesogenic activity was evaluated via their inhibitory activity on pancreatic lipase. Fermentation significantly altered the metabolite landscape, with 48 h identified as the optimal period for bioactive metabolite accumulation, depicted by enhanced phenolic, rare sugars, short chain fatty acids, carboxylic acids, amino acids, and nucleotides levels. Pathway enrichment revealed activation of relevant pathways for amino acid, nucleotide, and carboxylic acids metabolism. Antioxidant activity decreased with fermentation time, while enzyme inhibitory activities were enhanced. Molecular dynamics revealed potent molecular interactions of 2,6‐Dimethoxyphenol, filicinic acid, and pidolic acid with α‐glucosidase. The findings demonstrate that fermentation enhances the bioactive constituents of white corn steep liquor, with potent biological activities relevant to metabolic disease management.

## Graphical abstract

Time‐dependent fermentation caused dynamic changes in the bioactive constituents of white corn steep liquor and improved its antioxidant, antidiabetic, and antiobesogenic activities in vitro.

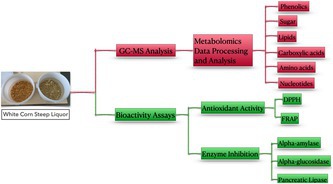

## 1 Introduction

Metabolic disorders represent some of the most widespread and challenging health conditions in modern clinical practice, often arising from disruptions in normal biochemical pathways triggered by poor dietary habits, physical inactivity, and a sedentary lifestyle (Tabatabaei‐Malazy et al. 2015). These disorders, whether inherited through rare genetic defects or acquired due to environmental and lifestyle factors, frequently coexist due to shared pathophysiological mechanisms such as insulin resistance, chronic inflammation, dyslipidemia, and gut dysbiosis. Acquired metabolic disorders include conditions such as obesity, type 2 diabetes, osteoporosis, hypertension, and non‐alcoholic fatty liver disease (Sethi and Hotamisligil 2021; Chooi et al. 2019). Their alarming rise in recent decades represents a significant global health burden, contributing substantially to morbidity, cancer risk, and premature mortality. Although lifestyle interventions like caloric restriction and increased physical activity remain foundational, pharmacological options for many of these conditions remain limited, underscoring the pressing need for innovative therapeutic approaches (Cousin et al. 2022; Toh et al. 2022).

Fermented foods and beverages have been integral to human diets for centuries, making up to 40% of some populations' intake. These products offer enhanced shelf life, safety, and organoleptic properties. Contemporary research highlights their potential health benefits, including improved nutritional value, weight maintenance, reduced cardiovascular disease risk, and enhanced immune function (C Borresen et al. 2012; Tamang et al. 2016). Through lactic acid fermentation, macronutrients are metabolized and anti‐nutritional factors degraded, enhancing digestibility, nutrient bioavailability, and functional properties (Sharma et al. 2020). Microorganisms achieve these transformations via enzymes such as proteases, peptidases, amylases, esterases, and phenol oxidases, converting substrates into palatable, nutritious products enriched with bioactive metabolites, including amino acids, peptides, exopolysaccharides, bacteriocins, and short‐chain fatty acids, that exhibit probiotic, antioxidant, and antimicrobial properties, thereby supporting gut health and modulating host metabolism (Nagarajan et al. 2022). These bioactive substances have been reported for their health benefits, including postbiotic activities, which are beneficial in the management of metabolic diseases (Marco et al. 2017; Taverniti and Guglielmetti 2011).

Recent studies suggest that fermented corn products, particularly corn steep liquor fermented with probiotic strains like *Lactobacillus acidophilus* and *Bifidobacterium brevis*, can yield compounds with antioxidant, immunomodulatory, and therapeutic potential (Susilowati et al. 2019). These include phenolics, organic acids, reducing sugars, and short chain fatty acids with documented beneficial effects on oxidative stress and inflammation. Such findings point to promising applications in managing metabolic dysfunction and gastrointestinal disorders (Wegh et al. 2019; Thorakkattu et al. 2022). However, there is still an incomplete understanding of their mechanisms of action, particularly regarding the specific bioactive small molecules that drive these functional benefits (Hijová 2024). To bridge this gap, metabolomics has emerged as a critical tool for characterizing the complex array of bioactive compounds generated during fermentation. Historically, the analysis of food components was limited to broad classifications such as carbohydrates, lipids, proteins, vitamins, minerals, and fiber. While informative, this traditional approach lacked the granularity required to fully capture the molecular intricacies of food. The advent of metabolomics has transformed this landscape, enabling researchers to profile foods and beverages with unprecedented molecular resolution. Today, it is known that a single food item can contain hundreds to thousands of distinct metabolites, each potentially influencing its functional and nutritional properties (Fraga‐Corral et al. 2022; Utpott et al. 2022). Building upon this foundation, the present study investigated the impact of crude fermentation on white corn steep liquor and evaluated its potential as a source of bioactive compounds relevant to metabolic disease management. Metabolomic approaches were employed to elucidate the biochemical dynamics and bioactive profiles generated during fermentation. Furthermore, the study assessed the effects of the liquors on key enzymes implicated in the progression of metabolic dysfunction.

## 2 Materials and Methods

### 2.1 White Corn Steep Liquor

White corn grains were obtained from a local market in Durban, South Africa. After manual sorting and thorough washing, 500 g of the grains were steeped in 1500 mL of distilled water at room temperature (25°C) for 7 days. Post‐steeping, the grains were rinsed, wet‐milled, and filtered to separate the fibrous residue from the starch‐rich slurry, following a previously described method (Okeke et al. 2018). The resulting slurry was then subjected to fermentation for 24, 48, and 72 h. After fermentation, the supernatants (steep liquor) were collected and freeze‐dried. The freeze‐dried samples were stored in sterile glass vials at room temperature for further analysis.

### 2.2 Derivatization

About 2 mg of the freeze‐dried samples was dissolved in 200 μL of distilled water in 2 mL Eppendorf tubes and mixed with 20 μL of N‐tert‐butyldimethylsilyl‐N‐methyltrifluoroacetamide (MTBSTFA) and N‐methyl‐N‐trimethylsilyl‐trifluoroacetamide (MSTFA). The reaction mixture was boiled for 30 min. The reaction mixture was allowed to cool and made to 1 mL with 99.9% ethanol.

### 2.3 Gas Chromatography–Mass Spectrometry (GC–MS) Analysis

The derivatized samples were subjected to GC–MS analysis using an Agilent technologies 6890 Series GC coupled with (an Agilent) 5973 Mass Selective detector (Agilent Technologies, Santa Clara, CA, USA) and driven by Agilent chemstation software. The metabolites were separated with a HP‐5MS capillary columns. 1 μL of the tissue supernatants was injected in a splitless mode. Carrier gas: ultra‐pure helium used as the carrier gas; Flow rate: 60 mL h^−1^; Initial oven temperature: 60°C for 2 min; Final oven temperature: 285°C at the rate of 5°C min^−1^; Hold time: 3 min; Ion source and quadrupole Temperatures: 230°C and 150°C, respectively; Electron ionization mode and electron multiplier voltage: 70 and 1859 V, respectively. The metabolites were identified by direct mass spectral comparison using an inbuilt NIST mass spectral library.

### 2.4 Pathway Analysis

The identified metabolites were subjected to pathway enrichment analysis using the MetaboAnalyst 6.0 online platform (https://www.metaboanalyst.ca/) as described by Pang et al. (2024).

### 2.5 Determination of pH Values

The pH values of the steep liquors were measured with an edge pH meter (Hanna Instruments, Woonsocket, RI, USA), according to the manufacturer's manual.

### 2.6 Determination of Antioxidant Activities

#### 2.6.1 2,2‐Diphenyl‐1‐Picrylhydrazyl (DPPH) Radical Scavenging Assay

The DPPH radical scavenging activity of the steep liquor samples was evaluated following a previously established protocol (Pobereżny et al. 2023). Briefly, 100 μL of each sample or standard solution (ascorbic acid, 15–240 μg/mL) was mixed with 50 μL of 0.3 mM DPPH solution prepared in methanol. The reaction mixtures were incubated in the dark at room temperature for 30 min. Absorbance was then measured at 517 nm, using a blank (containing methanol and DPPH without sample or standard) as the reference.

#### 2.6.2 Ferric Reducing Antioxidant Power (FRAP) Assay

The ferric reducing antioxidant power of the steep liquor samples was assessed based on a previously established method (Benzie and Strain 1996). Briefly, 100 μL of each sample or standard solution (ascorbic acid) was mixed with 100 μL of sodium phosphate buffer (0.2 M, pH 6.6) and 100 μL of 1% potassium ferricyanide. The mixture was incubated at 50°C for 30 min. Following incubation, 100 μL of 10% trichloroacetic acid (TCA) was added to terminate the reaction, followed by the addition of 100 μL of distilled water and 200 μL of 0.1% ferric chloride (FeCl₃). The absorbance was then measured at 700 nm using a microplate reader (SpectraMax M2, Molecular Devices, San Jose, CA, USA). The FRAP was expressed as a percentage of the sample's absorbance relative to that of gallic acid, calculated using the following formula:

$$\text{FRAP}\;\left(\%\right)=\left(\text{Absorbance of sample}/\text{Absorbance of gallic acid}\right)\times 100$$

### 2.7 Inhibitory Effect on Digestive Enzymes

#### 2.7.1 α‐Glucosidase Inhibition Assay

The in vitro α‐glucosidase inhibitory potential of the steep liquor samples was evaluated following the method described by Ademiluyi and Oboh (2013), with minor modifications. Briefly, 50 μL of each steep liquor sample was mixed with 50 μL of α‐glucosidase solution (1.0 U/mL) prepared in 100 mM phosphate buffer (pH 6.8) and incubated at 37°C for 15 min. After incubation, 100 μL of 5 mM p‐nitrophenyl‐α‐D‐glucopyranoside (pNPG) in the same buffer was added to initiate the reaction, followed by an additional 20‐min incubation at 37°C. The release of p‐nitrophenol was quantified by measuring absorbance at 405 nm with a microplate reader (SpectraMax M2, Molecular Devices, San Jose, CA, USA). The percentage inhibition was determined relative to a control (without inhibitor) using the following formula:

$$\%\text{inhibition}=\left[\left(\text{Absorbance of control}-\text{Absorbance of sample}\right)/\text{Absorbance of control}\right]\times 100$$

Acarbose was used as the reference antidiabetic agent.

#### 2.7.2 α‐Amylase Inhibition Assay

The ability of the white corn steep liquor samples to inhibit α‐amylase activity was assessed based on the method outlined by Shai et al. (2010) with slight modifications. Briefly, 50 μL of each steep liquor sample or acarbose (standard inhibitor) was mixed with 50 μL of porcine pancreatic α‐amylase (2 U/mL) in 100 mM phosphate buffer (pH 6.8), and the mixture was incubated at 37°C for 10 min. Next, 50 μL of 1% soluble starch solution prepared in the same buffer was added to initiate the enzymatic reaction, followed by another 10‐min incubation at 37°C. The reaction was terminated by adding 100 μL of dinitrosalicylic acid (DNS) reagent, and the mixture was then heated in boiling water for 10 min. The absorbance of the resulting solution was measured at 540 nm with a microplate reader (SpectraMax M2, Molecular Devices, San Jose, CA, USA). The percentage inhibition was calculated relative to a control sample without inhibitors using the formula:

$$\%\text{inhibition}=\left[\left(\text{Absorbance of control}-\text{Absorbance of sample}\right)/\text{Absorbance of control}\right]\times 100$$

#### 2.7.3 Pancreatic Lipase Inhibition Assay

The in vitro ability of the white corn steep liquor samples to inhibit pancreatic lipase was evaluated using a modified version of the method described by Kim et al. (2010). Briefly, 50 μL of each steep liquor sample or orlistat (standard drug) was mixed with 84.5 μL of Tris buffer (100 mM Tris–HCl with 5 mM CaCl_2_, pH 7.0). Then, 20 μL of porcine pancreatic lipase (2.5 mg/mL prepared in 10 mM MOPS and 1 mM EDTA, pH 6.8) was added to the mixture and incubated at 37°C for 15 min. The reaction was initiated by adding 5 μL of 10 mM p‐nitrophenyl butyrate (p‐NPB) dissolved in dimethyl formamide, followed by a further incubation at 37°C for 30 min. Absorbance was read at 405 nm using a microplate reader (SpectraMax M2, Molecular Devices, San Jose, CA, USA), and lipase inhibition was calculated as a percentage relative to the control (without inhibitor) using the formula:

$$\%\text{inhibition}=\left[\left(\text{Absorbance of control}-\text{Absorbance of sample}\right)/\text{Absorbance of control}\right]\times 100$$

### 2.8 Computational Studies

#### 2.8.1 Molecular Docking

The following identified metabolites comprising 2,6‐Dimethoxyphenol, filicinic acid, pidolic acid, o‐guaiacol, tyrosol, methyl beta‐d‐ribopyranoside, cysteine, lactic acid, and 4‐hydroxybutanoic acid were subjected to molecular docking analysis with α‐glucosidase. The 3D structures of the enzyme (PDB ID: 3L4Y) (Sim et al. 2010) were retrieved from the Protein Data Bank (PDB) and prepared using UCSF Chimera software (Shai et al. 2010). MarvinSketch 6.2.1, Molegro Molecular Viewer (MMV), and ChemAxon were utilized in optimizing the compounds for correct hybridization states and molecular geometry (Mohamed et al. 2025). The Desmond software (Schrödinger 2023–2) was utilized in performing docking stimulation with OPLS‐2005 force field. BIOVIA Discovery Studio Visualizer was used in visualizing and analyzing the ligand‐enzyme complexes. Ligand‐protein complexes with top scoring energies were selected for molecular dynamics simulation.

#### 2.8.2 Molecular Dynamics Simulation

Molecular dynamics simulation (MDS) was carried out to determine the stability and dynamic behavior of the selected ligand‐protein complexes using the Desmond module of Schrödinger 2023–2. It was run remotely at the Centre for High Performance Computing (CHPC), Cape Town, South Africa. Briefly, the respective complexes were placed in an orthorhombic box with a single‐point charge (SPC) to prepare them for MDS, within a buffer distance of 10 Å. The transferable intermolecular potential 3P (TIP3P) water model was used in solvating the system and was neutralized with 0.15 M NaCl and Na^+^/Cl^−^ ions. The particle‐mesh Ewald method was used in calculating the long‐range electrostatic interactions, while short‐range van der Waals and Coulomb interactions were truncated at a radius of 9.0 Å. The solvated system was minimized with the OPLS‐2005 forcefield (2023) parameters and relaxation thereafter (Bowers et al. 2006). The system was stimulated with Berendsen NVT by maintaining the pressure and temperature at 1.01325 bar and temperature 300 K with Nosè–Hoover chain thermostat and Martyna–Tobias–Klein barostat, respectively (Martyna et al. 1994). The NPT ensemble was initiated with a production run lasting 100 ns and measured at every 50 ps as the trajectories progressed. The resulting trajectories which consisted of protein stability (RMSD) and flexibility (RMSF) were measured with an AMBER 20 integrated CPPTRAJ module (Erukainure et al. 2024).

### 2.9 Statistical Analysis

Multivariate statistical analysis, including clustering analysis for heatmaps and principal component analysis (PCA), was performed using the MetaboAnalyst 6.0 online server (https://www.metaboanalyst.ca/) (Pang et al. 2024).

All biological assays were performed in triplicates, and data were statistically analyzed using SPSS software (version 27, International Business Machines (IBM) Corporation, Armonk, NY, USA). One‐way ANOVA was employed to assess the results and expressed as mean ± standard deviation (SD). *p*‐value of less than 0.05 was considered statistically significant using the Tukey's HSD‐multiple range post hoc test.

## 3 Results and Discussion

Phytoconstituents and other products from fermentation have been explored in the folk medicine for the management of various ailments including metabolic diseases. Corn starch liquor, a product of traditional lactic acid fermented corn starch, has been reported for its rich source of organic acids, reducing sugars, lipids, enzymes, phenolics, and other functional components such as phenolics (Chang et al. 2025; Zhou et al. 2022). These constituents have been reported for their medicinal properties, which contribute to the overall therapeutic effects of the liquor on metabolic diseases including diabetes (Karigidi and Olaiya 2020). In the present study, we investigated the dynamic changes in pH values, phytochemicals, nutritional and organic acid constituents associated with fermentation time of corn starch liquor. Pathways involved in these dynamics were further elucidated via pathway enrichment.

### 3.1 Phenolic Constituents

**FIGURE 1:**
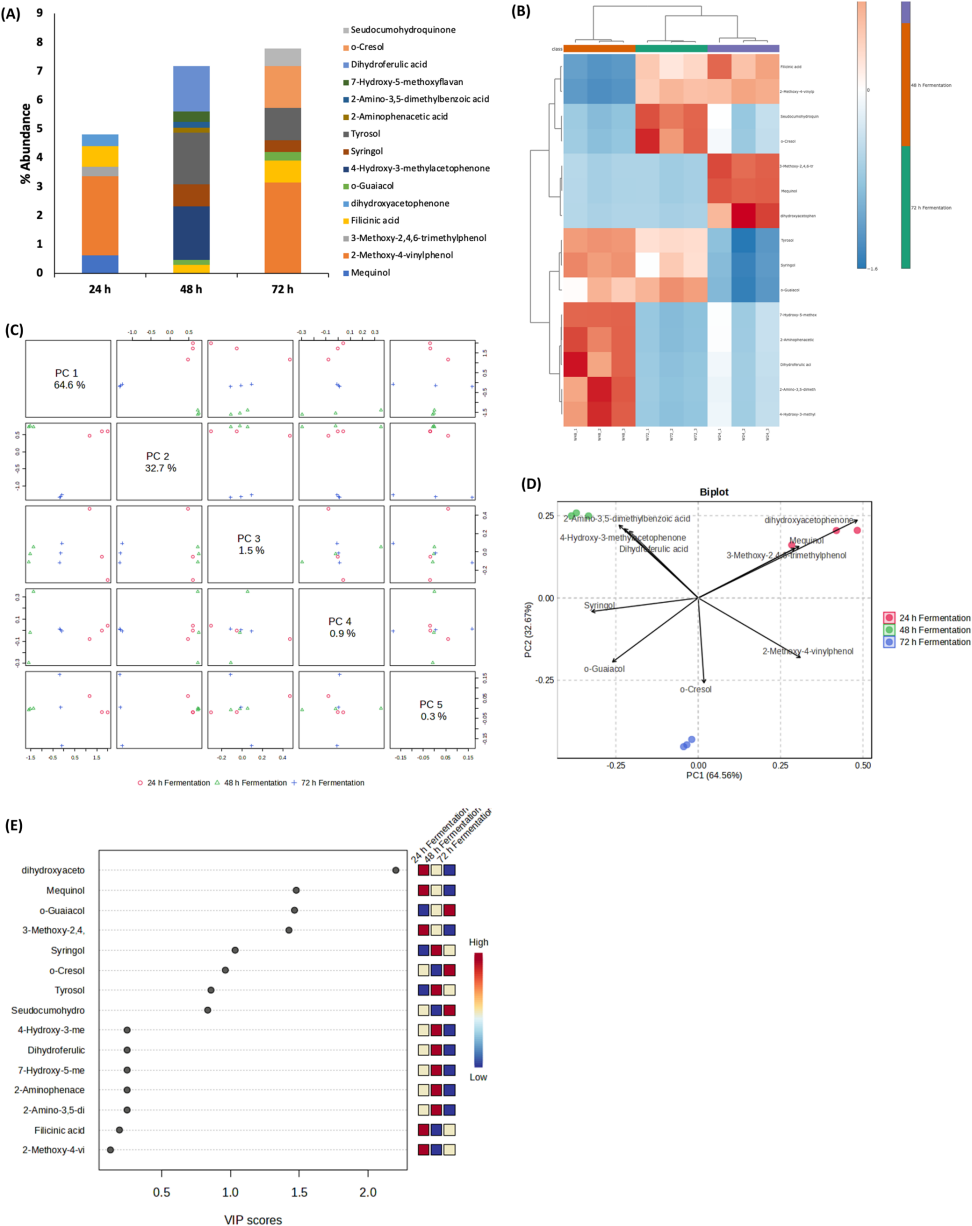
Effect of fermentation on phenolic constituents of white corn steep liquor. (A) Phenolic constituents; (B) heatmap distribution; (C) PC scores; (D) biplot; and (E) VIP scores.

Phenolics play a key role in the antioxidant and anti‐inflammatory properties of plants, with established effects on oxidative stress regulation, inflammatory pathways, and glucose metabolism (Liu et al. 2023). Fermentation enhances the bioavailability and bioactivity of polyphenols, with microorganisms such as lactic acid bacteria and yeasts transforming complex polyphenols into simpler, more soluble forms, increasing total phenolic content and antioxidant activities (Sharma et al. 2022). As shown in Figure 1, quantification and multivariate analysis revealed fermentation‐induced shifts in phenolic profiles of the steep liquor. Notably, dihydroxyacetophenone, 3‐methoxy‐2,4,6‐trimethylphenol, and syringol increased significantly after 48 h, as evidenced in panel (Figure 1A). The heatmap (Figure 1B) and PCA plot (Figure 1C) highlighted the 48‐h fermentation stage as the point of greatest compositional divergence, confirmed by biplot (Figure 1D) clustering and VIP scores (Figure 1E), where dihydroxyacetophenone contributed the highest discriminatory power. This enhanced phenolic profile suggests stronger antioxidant, anti‐inflammatory, and metabolic regulatory potential, as dihydroxyacetophenone isomers, among other phenolics, exhibit diverse bioactivities, including anti‐inflammatory, endothelial‐protective, and glucose‐ and lipid‐lowering effects (Yang et al. 2023). Thus, indicating the potential of the steep liquor in metabolic disease management, with fermentation playing an influential role.

### 3.2 Sugar Constituents

**FIGURE 2:**
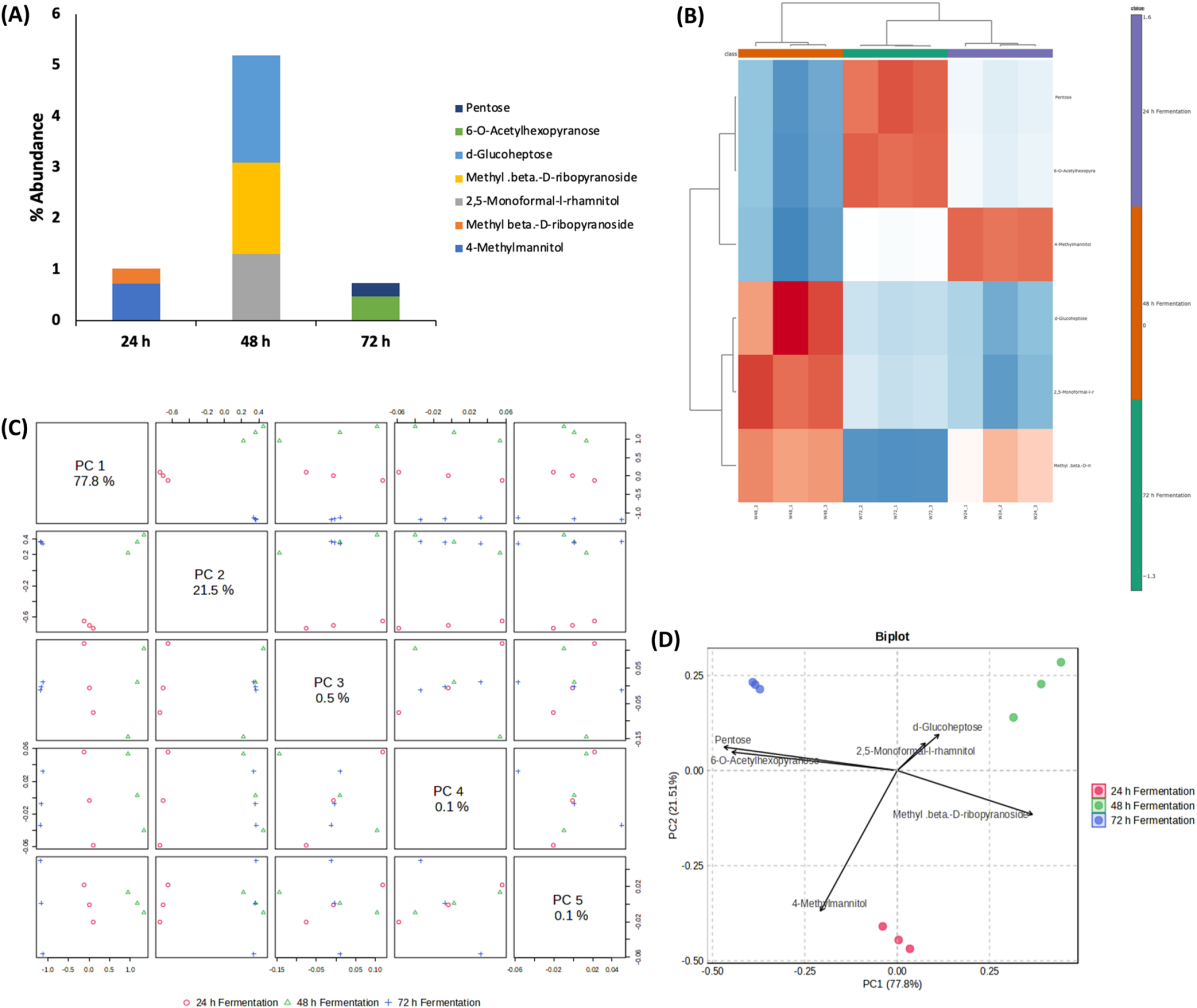
Effect of fermentation on sugar constituents of white corn steep liquor. (A) Sugar constituents; (B) Heatmap distribution; (C) PC scores; and (D) Biplot.

Probiotic bacteria primarily utilize carbohydrates for energy, converting them into organic acids through fermentation. These organic acids, including lactic, acetic, and propionic acids, create an unfavorable environment for pathogenic microorganisms and contribute to food preservation (Bangar et al. 2022). These microbes, particularly *Lactobacillus* and *Bifidobacterium* species, possess a diverse array of carbohydrate‐metabolizing enzymes and transport systems that enable them to utilize various oligosaccharides, including host‐derived glycans (Zúñiga et al. 2018). These bacteria express glycoside hydrolases, sugar ABC transporters, and phosphoenolpyruvate‐dependent phosphotransferase systems for the uptake and catabolism of prebiotic compounds (Andersen et al. 2012). Fermentation of white corn steep liquor significantly altered its sugar profile in a time‐dependent manner. As shown in Figure 2A, there was a slight increase in 4‐methylmannitol and methyl‐β‐D‐ribopyranoside at 24 h, followed by significant accumulation of D‐glucoheptose, methyl‐β‐D‐ribopyranoside, and 2,5‐monoformal‐L‐rhamnitol at 48 h, suggesting peak metabolic conversion and rare sugar production. At 72 h, there was a moderate increase in 6‐O‐acetylhexopyranose and pentoses, possibly reflecting late‐stage polysaccharide degradation. The heatmap (Figure 2B) and PCA plots (Figure 2C,D) depicted clear temporal clustering, indicating progressive biochemical divergence. The depletion of fermentable sugars alongside the emergence of structurally diverse carbohydrate derivatives suggests active microbial transformation and postbiotic formation, potentially lowering glycaemic index and enhancing the metabolic health benefits of the fermented product (van Hylckama Vlieg et al. 2011).

### 3.3 Lipid Constituents

**FIGURE 3:**
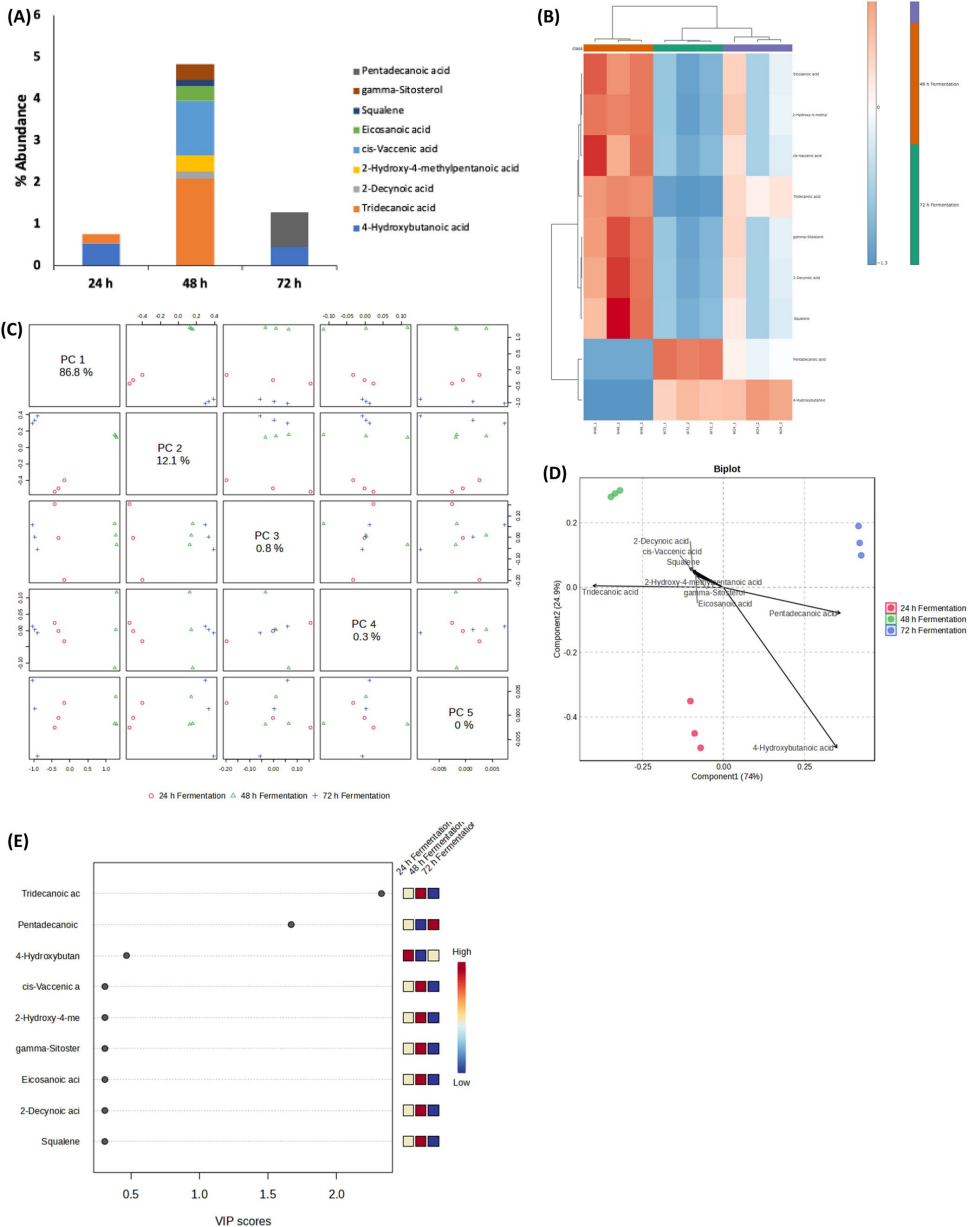
Effects of fermentation on lipid constituents of white corn steep liquor (A) Lipid constituents; (B) Heatmap distribution; (C) PC scores; (D) Biplot; and (E) VIP scores.

Probiotics, particularly lactic acid bacteria, have been shown to influence lipid metabolism through various mechanisms. Fermentation alters the profiles of both short‐ and long‐chain fatty acids, including butyric, oleic, palmitic, and conjugated linoleic acids. These changes are mainly due to the lipolytic and proteolytic enzyme activities of certain probiotic bacteria (Feng et al. 2021). As shown in Figure 3A, fermentation significantly increased tridecanoic acid and pentadecanoic acid levels after 48 h and 72 h, respectively. These shifts were further validated through heatmap analysis, PCA, and VIP scores (Figure 3B–E), reflecting distinct lipidomic remodeling. Pentadecanoic acid has demonstrated insulin‐sensitizing, anti‐inflammatory, and hepatoprotective effects (Ciesielski et al. 2024). Though traditionally considered markers of dairy fat intake, saturated fatty acids may also arise endogenously and contribute to metabolic regulation via anaplerotic support of the citric acid cycle (Pfeuffer and Jaudszus 2016). Their upregulation suggests that time‐dependent fermentation may improve the postbiotic properties of corn steep liquor which are relevant to metabolic disease prevention and gut health modulation.

### 3.4 Carboxylic Acids Constituents

**FIGURE 4:**
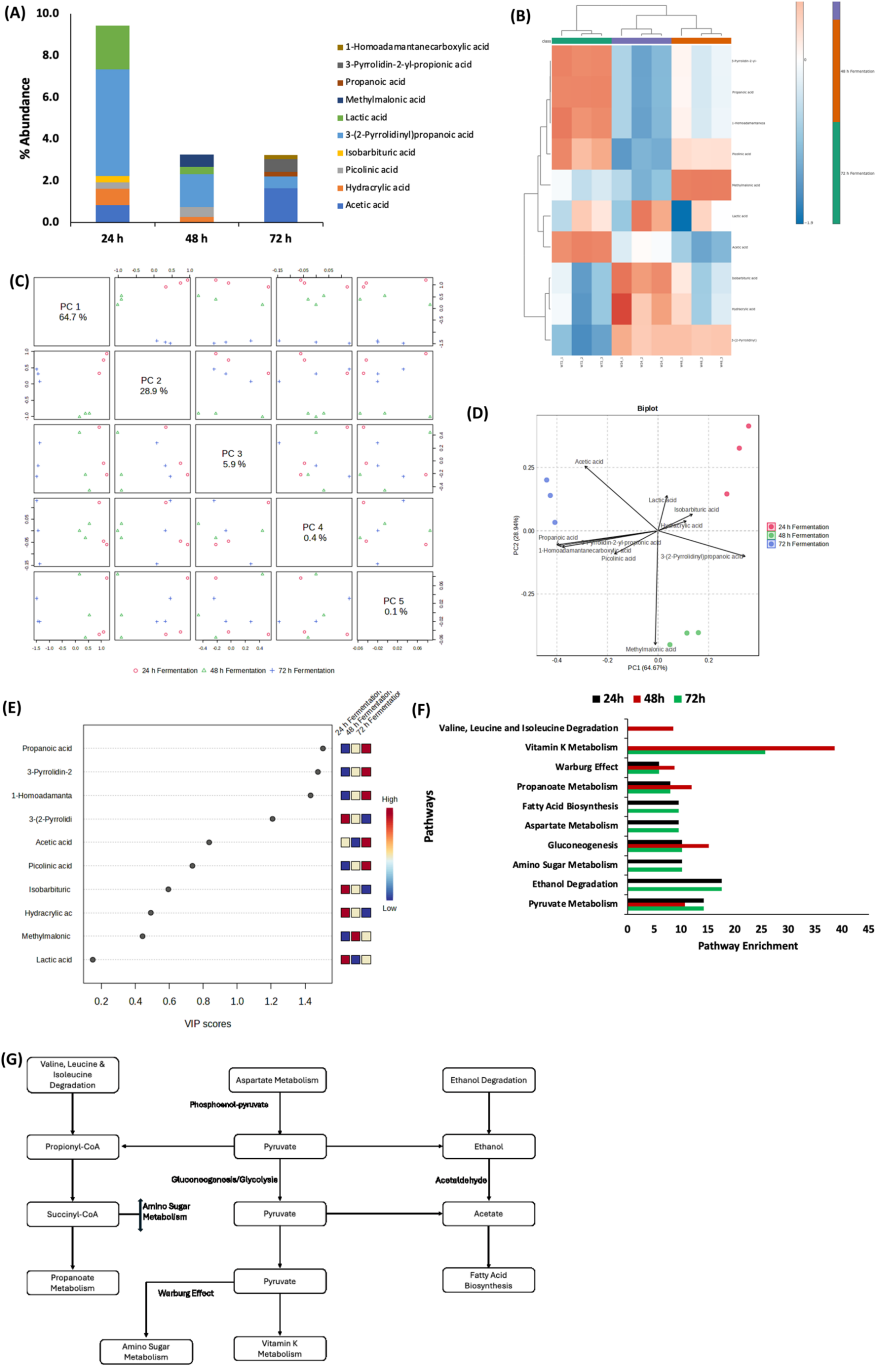
Effect of fermentation on carboxylic acids constituents of white corn steep liquor. (A) Carboxylic constituents; (B) Heatmap distribution; (C) PC scores; (D) Biplot; (E) VIP scores; (F) Pathway enrichment of identified metabolites; and (G) Metabolic map of identified pathways.

Carboxylic acids, produced through fermentation, are valuable chemicals with diverse health and industrial applications (López‐Garzón and Straathof 2014). They demonstrate antioxidant and anti‐inflammatory properties by activating the nuclear factor erythroid 2–related factor 2 (Nrf2) pathway, potentially treating oxidative‐mediated diseases (Egbujor 2024). Their antimicrobial properties have long been exploited in food preservation and wound treatment (Mira et al. 2024). As shown in Figure 4A, fermentation promoted a significant accumulation of carboxylic acids with strong clustering at 72 and 48 h, notably propanoic acid and its derivatives (3‐pyrrolidin‐2‐yl‐propionic acid, 3‐(pyrrolidinyl) propanoic acid), 1‐homoadamantane carboxylic acid, and acetic acids, core metabolic intermediates in energy metabolism and gut microbiota modulation. Heatmap distribution and PCA (Figure 4B–D) revealed a fermentation‐time‐dependent shift, while VIP scores (Figure 4E) highlighted propanoic acid and 1‐homoadamantane carboxylic acid as major contributors. Pathway enrichment (Figure 4F) and metabolic mapping (Figure 4G) indicated increased activity in gluconeogenesis, pyruvate metabolism, and propanoate metabolism pathways during fermentation, reflecting active substrate utilization and metabolic adaptation by the fermenting microorganisms. The accumulation of these organic acids is closely tied to fermentation dynamics and is shaped by fermentation time, which governs microbial activity, substrate conversion, and shifts in dominant metabolic pathways (Krishnan et al. 2015).

### 3.5 Amino Acids Constituents

**FIGURE 5:**
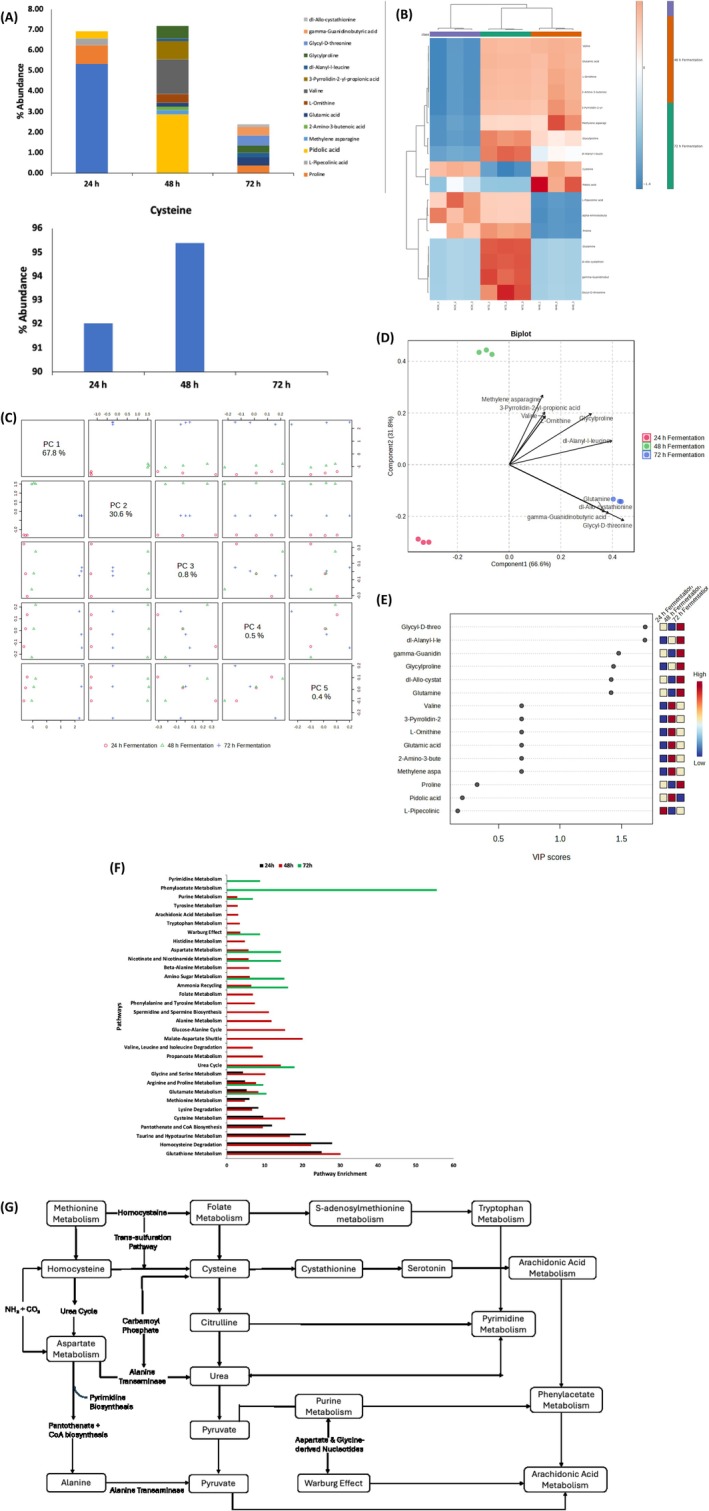
Effect of fermentation on amino acids constituents of white corn steep liquor. (A) Amino acids constituents; (B) Heatmap distribution; (C) PC scores; (D) Biplot; (E) VIP scores; (F) Pathway enrichment of identified metabolites; and (G) Metabolic map of identified pathways.

Fermentation enhances the nutritional profile of corn and its products by increasing protein content, amino acid levels, and total phenolics while reducing anti‐nutritional factors such as phytic acid (Cui et al. 2012). Amino acids are pivotal for metabolic health, regulating glucose and lipid metabolism, energy balance, and mitochondrial biogenesis (Bifari et al. 2017). Additionally, amino acids and their metabolites modulate glycolipid metabolism by influencing key signaling pathways and shaping gut microbial composition (Xie et al. 2020). Fermentation substantially modulated amino acid concentrations (Figure 5A–B), with notable increases in glycyl‐D‐threonine, dl‐Alanyl‐l‐leucine, gamma‐guanidinobutyric acid, glycylproline, and dl‐Allo‐cystathionine after 72 h. These changes may reflect both proteolytic activity and microbial biosynthesis, yielding metabolites that support the intestinal barrier, regulate immune responses, and influence gut microbiota composition (Luise et al. 2023). PCA and biplot clustering (Figure 5C,D) demonstrated a time‐dependent amino acid shift, supported by VIP rankings (Figure 5E). Pathway enrichment (Figure 5F) indicated that the fermenting microorganisms actively utilize glutathione metabolism, homocysteine degradation, and taurine/hypotaurine metabolism pathways during fermentation. These pathways (Figure 5G) reflect the metabolic dynamics of the fermentation process, which is influenced by fermentation time and microbial activity (Brosnan and Brosnan 2006). The resulting metabolites produced through these pathways constitute potential postbiotic compounds, collectively enhancing the nutritional and functional profile of the fermented liquor.

### 3.6 Nucleotide Constituents

**FIGURE 6:**
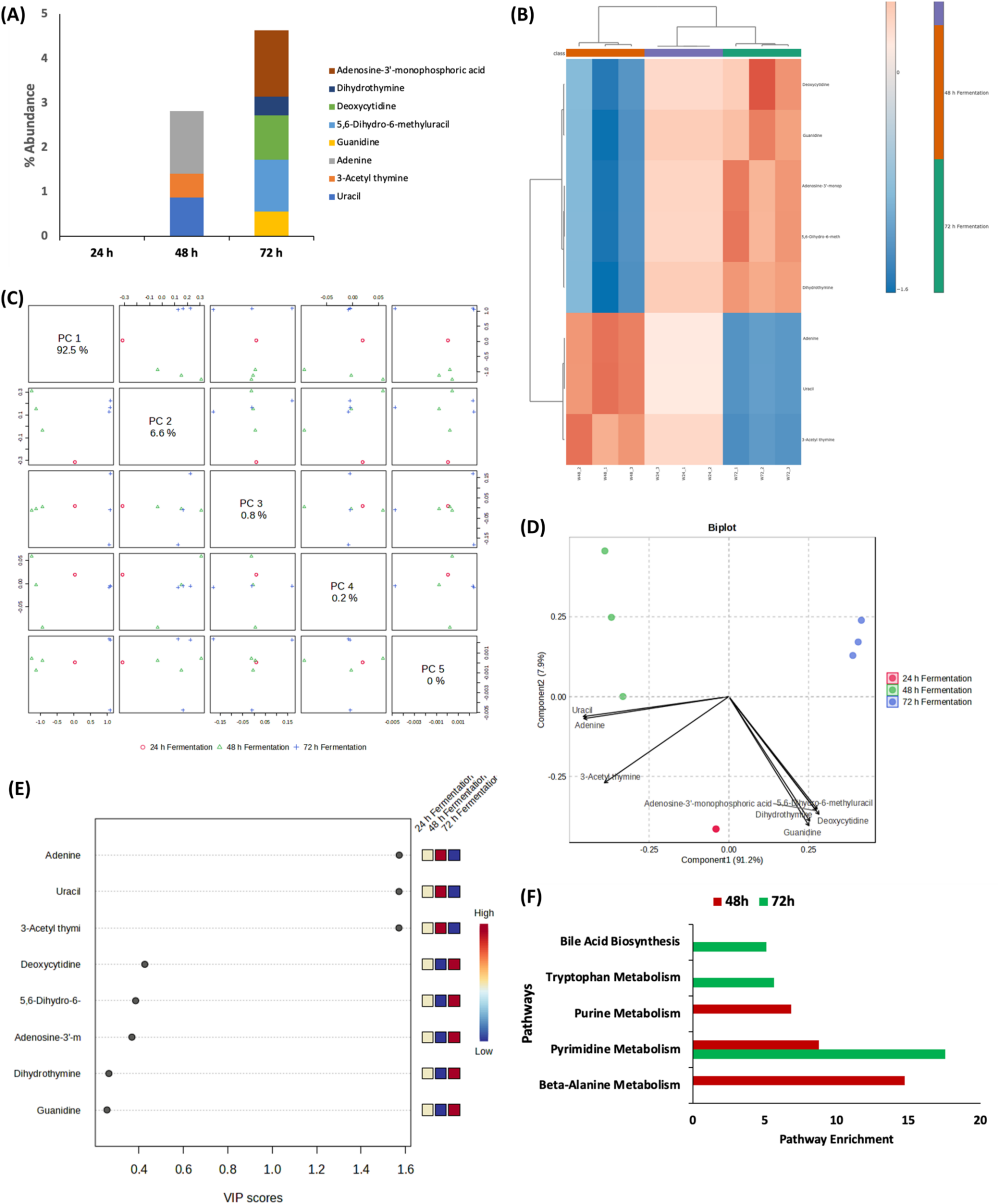
Effect of fermentation on nucleotide constituents of white corn steep liquor. (A) Nucleotide constituents; (B) Heatmap distribution; (C) PC scores; (D) Biplot; (E) VIP scores; and (F) Pathway enrichment of identified metabolites.

Nucleotides are vital for maintaining immune health, promoting intestinal maturation, and facilitating recovery under stress or pathological conditions. While endogenous production is the primary source, dietary nucleotides are considered conditionally essential during periods of rapid growth, injury, or disease (Hess and Greenberg 2012). Their accumulation indicates nucleotide salvage and biosynthesis activity. Fermentation influenced nucleotide composition (Figure 6A), with elevated levels of adenine, thymine, and uracil: components involved in cellular signaling, energy transfer, and immune modulation. Multivariate analysis (Figure 6B–E) confirmed distinct clustering after 48 h, while pathway enrichment analysis (Figure 6F) revealed activation of pyrimidine and purine metabolism, beta‐alanine metabolism, tryptophan metabolism, and bile acid biosynthesis. These pathways collectively reflect the metabolic activity of fermenting microorganisms, indicating enhanced nucleotide turnover, biogenic amine and nicotinamide metabolism, antioxidant buffering, and lipid processing during fermentation (D'Mello 2003; Bakker and Bierau 2012). Such shifts illustrate the fermentation dynamics within the liquor, driven by microbial metabolism and fermentation conditions, which in turn determine the profile of metabolites with potential postbiotic relevance.

### 3.7 Changes in pH Values

**FIGURE 7:**
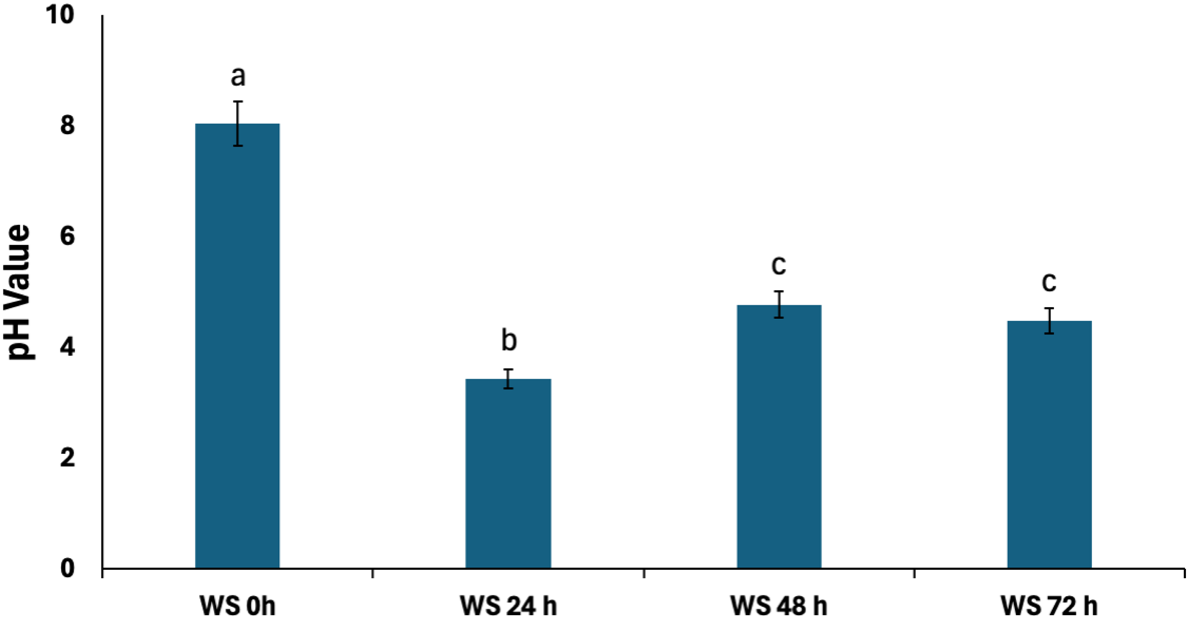
Effect of fermentation on the pH value of white corn steep liquor. Values = mean ± SD; *n* = 3. (a–c) Values with different letters above the bars are significantly different from each other (*p* < 0.05, Tukey's HSD‐multiple range post hoc test, IBM SPSS for Windows).

Changes in pH value towards acidic level is a major characteristic of fermentation and has been reported in corn starch liquor (Chang et al. 2025; Liu et al. 2024). As depicted in Figure 7, there was a significant (*p* < 0.05) drop in pH level following 24 h fermentation, indicating increased acid concentration. This change can be attributed to increased production of organic acids from nutrients consumed by microorganisms (Jabłońska‐Ryś et al. 2022; Thuy et al. 2024; Wang et al. 2005). This is further corroborated by the high concentrations of carboxylic acids in 24 h fermented liquor (Figure 4A). As fermentation time increased, the pH values were observed to increase. This can be attributed to the breakdown of the generated organic acids, leading to their decreased concentration and increased alkalinization (Calsamiglia et al. 2002; Ozoh et al. 2023; Estoppey et al. 2025).

### 3.8 Antioxidant Activities

**FIGURE 8:**
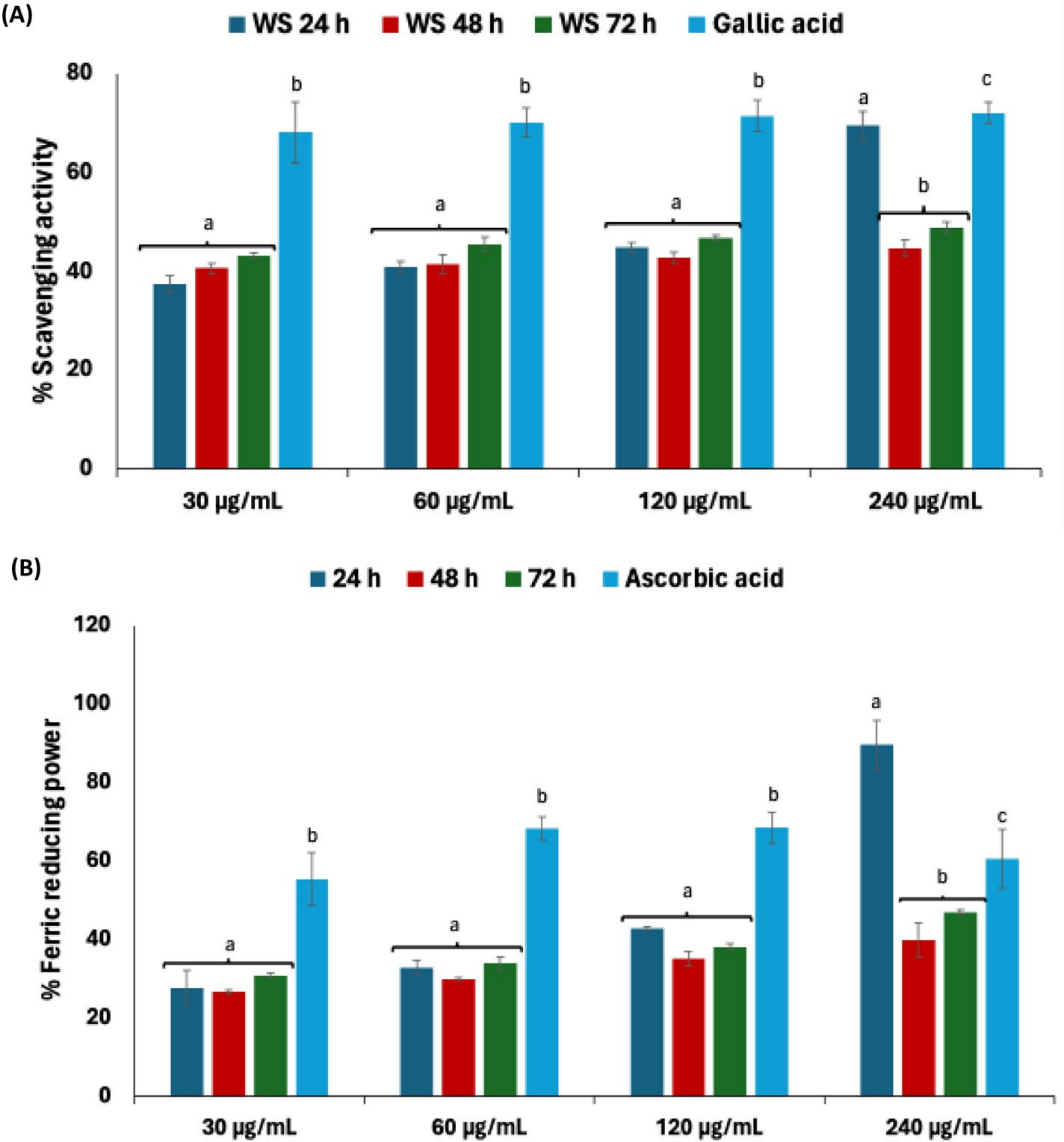
Effect of fermentation on (A) DPPH scavenging activity and (B) ferric reducing power of white corn steep liquor. Values = mean ± SD; *n* = 3. ^a,b^Values with different letters above the bars are significantly different from each other (*p* < 0.05, Tukey's HSD‐multiple range post hoc test, IBM SPSS for Windows).

The antioxidant activities of plant extracts are commonly evaluated using assays such as DPPH radical scavenging and FRAP which have been widely applied across various plant species (Clarke et al. 2013). As indicated in Figure 8, there were significant (*p* < 0.05) dose‐dependent increases in the DPPH scavenging activity and FRAP of the steep liquor. However, these activities declined with increasing fermentation time, with 24 h exhibiting the highest activity as depicted by their IC_50_ values (DPPH = 96.26 μg/mL; FRAP = 90.33 μg/mL) (Table 1). These outcomes can be attributed to the synergistic actions of the identified metabolites, particularly phenolics. The findings indicate that controlled fermentation can enhance the functional value of white corn steep liquor as a potential antioxidant source. Consistent with this, other studies have shown that fermenting corn by‐products with lactic acid bacteria, notably *L. plantarum* and *P. pentosaceus* , significantly increases antioxidant activity (Tonolo et al. 2023).

**TABLE 1:** IC_50_ values of biological activities.

Treatments	DPPH	FRAP	α‐glucosidase	α‐amylase	Lipase
	(μg/mL)				
WS 24 h	96.26	90.33	2.39	> 1000	1.12
WS 48 h	> 1000	> 1000	0.59	> 1000	1.00
WS 72 h	379.40	444.65	1.87	691.60	1.00
Gallic acid	15.54	NA	NA	NA	NA
Ascorbic acid	NA	4.33	NA	NA	NA
Acarbose	NA	NA	70.6.	30.88	NA
Orlistat	NA	NA	NA	NA	1.30

Abbreviation: NA, not applicable.

### 3.9 Enzyme Inhibitory Activities

**FIGURE 9:**
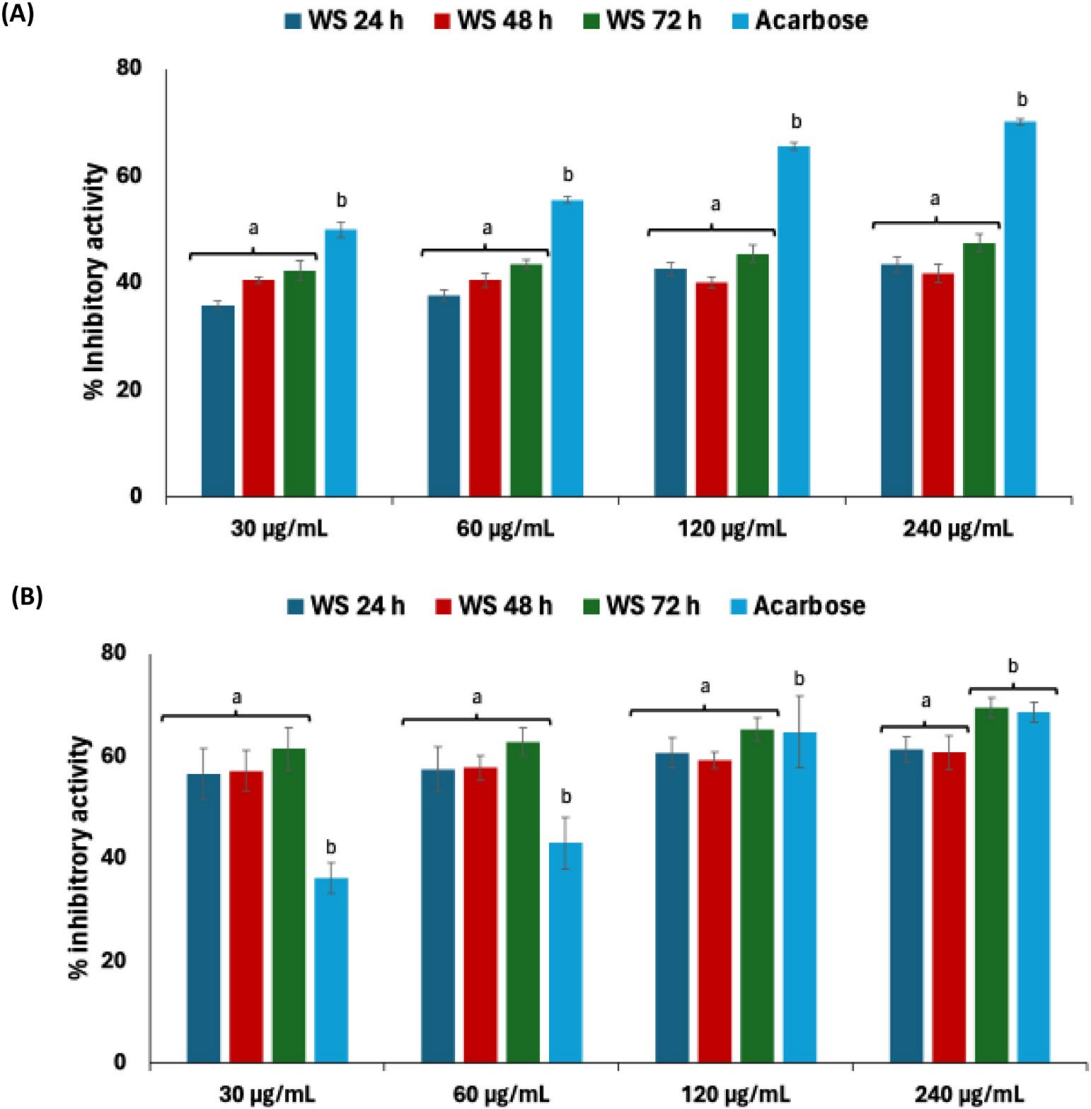
Effect of fermentation of (A) α‐amylase and (B) α‐glucosidase inhibitory activities of white corn steep liquor. Values = mean ± SD; *n* = 3. ^a,b^Values with different letters above the bars are significantly different from each other (*p* < 0.05, Tukey's HSD‐multiple range post hoc test, IBM SPSS for Windows).

Natural inhibitors of α‐amylase and α‐glucosidase have attracted interest for their role in regulating blood glucose levels in diabetic management (Tundis et al. 2010). As shown in Figure 9A, the fermentation of white corn steep liquor exhibited relatively weak α‐amylase inhibitory activity across all fermentation periods (IC_50_ > 1000 μg/mL), indicating minimal interference with complex carbohydrate digestion, an advantage for reducing gastrointestinal side effects commonly associated with strong α‐amylase inhibitors. Corn‐derived compounds have shown promising α‐glucosidase inhibitory activity for managing postprandial hyperglycaemia (Lee et al. 2025), a finding supported by the strong inhibition following 48 h fermentation (IC_50_ = 0.59 μg/mL) (Figure 9B), which outperformed acarbose (IC_50_ = 70.6 μg/mL) and suggests robust potential for postprandial glycaemic control.

**FIGURE 10:**
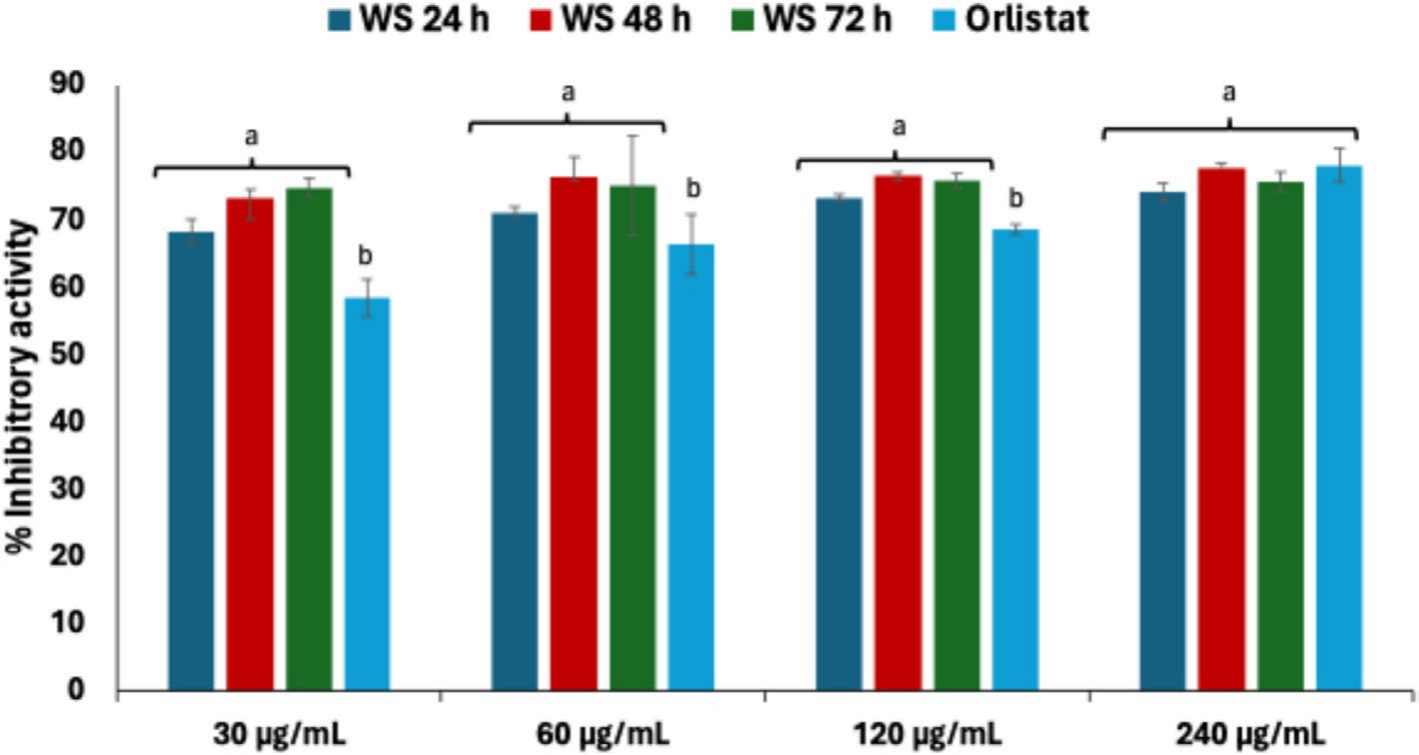
Effect of fermentation on lipase inhibitory activity of white corn steep liquor. Values = mean ± SD; *n* = 3. (a, b) Values with different letters above the bars are significantly different from each other (*p* < 0.05, Tukey's HSD‐multiple range post hoc test, IBM SPSS for Windows).

Lipase inhibition has gained attention as a strategy for treating obesity and related disorders by suppressing the hydrolysis of dietary lipids (Singh et al. 2015). A potent and consistent inhibition of pancreatic lipase was observed with increasing fermentation time (Figure 10), particularly at 48 and 72 h (IC_50_ = 1.00 μg/mL), significantly surpassing orlistat (IC_50_ = 1.30 μg/mL). This finding highlights the antiobesogenic potential of the fermented liquor through delayed lipid hydrolysis, relevant in obesity and dyslipidaemia management (Bülbül and Çokdinleyen 2024).

### 3.10 Molecular Interactions

**FIGURE 11:**
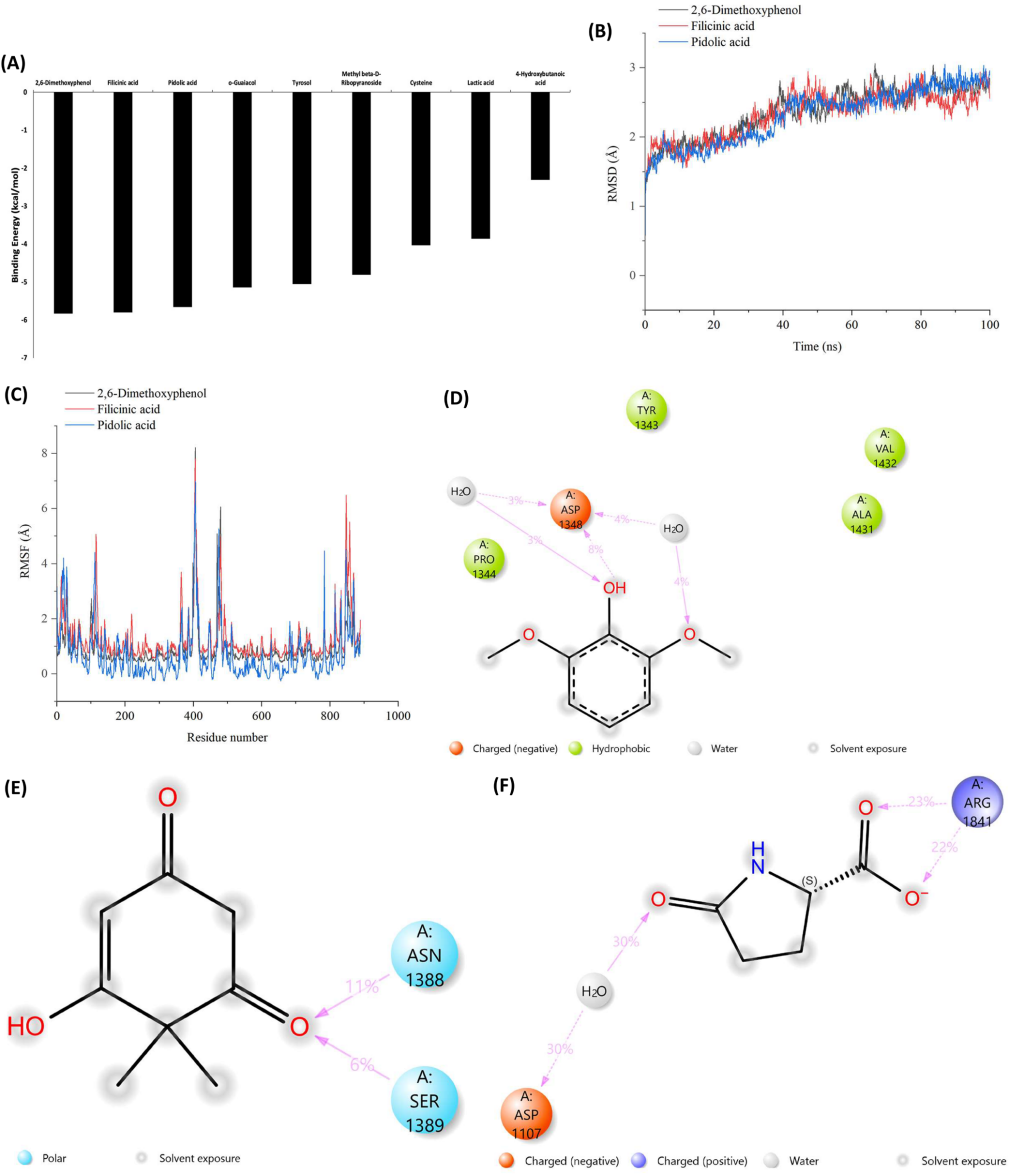
Molecular docking and dynamics of selected metabolites of white corn steep liquor with α‐glucosidase. (A) Binding energies; (B) RMSD (Å); (C) RMSF (Å) values; and ligand‐protein interactions of (D) 2,6‐Dimethoxyphenol, (E) filicinic acid, and (F) pidolic acid with α‐glucosidase.

To understand the antidiabetic mechanism of the identified metabolites, particularly the phenolics, we investigated their molecular interactions with α‐glucosidase using computational tools. The choice of α‐glucosidase was based on the IC_50_ value. As shown in Figure 11A, molecular docking analysis revealed potent interaction between the studied metabolites and the enzyme, with 2,6‐Dimethoxyphenol, filicinic acid, and pidolic acid being the most potent as depicted by their low binding energies. In molecular docking studies, low binding energies suggest a stronger and more favorable interaction between a protein and a ligand (Alsedfy et al. 2024). The stability conformation of the ligand‐protein complexes was further confirmed by the RMSD values (Figure 11B and Table 2). This was further supported by the RMSF values, which indicated the rigidity of the ligand‐protein complexes, with 2,6‐Dimethoxyphenol being the most rigid (Figure 11C and Table 2). RMSD and RMSF measure the stability and rigidity of molecular interactions in ligand‐protein complex (Kumar et al. 2024; Martínez 2015; Bagewadi et al. 2023). Thus, giving more credence to the antidiabetic potentials of the liquors. The major molecular bond contributors were positive charges, hydrophobic, solvent exposure, and water bridges for 2,6‐Dimethoxyphenol (Figure 11D); polar and solvent exposure for filicinic acid (Figure 11E); and positive and negative charges, solvent exposure, and water bridges for pidolic acid (Figure 11F).

**TABLE 2:** RMSD and RMSF profile of 2,6‐Dimethoxyphenol, filicinic acid, and pidolic acid bound to α‐glucosidase.

Compounds	RMSD (Å)	RMSF (Å)
2,6‐Dimethoxyphenol	2.36	0.92
Filicinic acid	2.32	1.03
Pidolic acid	2.31	1.02

### 3.11 Study Limitations

The present study did not identify the microorganisms responsible for the fermentation. However, studies are ongoing to identify and characterize the fermenting microorganisms.

## 4 Conclusion

This study gives preliminary insights into the dynamic biochemical transformations during crude fermentation of white corn steep liquor, revealing its potential as a valuable source of phytochemicals, organic acids and nutrients suitable for the management of metabolic diseases. The identified bioactive compounds, antioxidant, antihyperglycemic, and antiobesogenic activities improved with increasing fermentation time. These insights may provide a foundation for advancing fermented corn steep liquor as a functional ingredient in therapeutic dietary interventions targeting obesity, type 2 diabetes, and related metabolic disorders. However, further studies are warranted to identify the fermenting microorganisms, as well as decipher these in vitro findings in in vivo models of metabolic diseases as well as clinical trials.

## Author Contributions

**Lemohang Gumenku:** investigation, writing – original draft, formal analysis, methodology. **S'thandiwe N. Magwaza:** investigation, methodology, writing – review and editing. **Ochuko L. Erukainure:** conceptualization, investigation, writing – original draft, writing – review and editing, methodology, project administration, supervision. **Ademola O. Olaniran:** funding acquisition, writing – review and editing, validation, supervision, resources, project administration. **Md. Shahidul Islam:** funding acquisition, writing – review and editing, supervision, resources, project administration. **Almahi I. Mohamed:** investigation, methodology, software, writing – original draft.

## Funding

This work was supported by Inyuvesi Yakwazulu‐Natali, 224198855.

## Conflicts of Interest

The authors declare no conflicts of interest.

## Data Availability

The data that support the findings of this study are available on request from the corresponding author. The data are not publicly available due to privacy or ethical restrictions.

## References

[fsn371884-bib-0001] Ademiluyi, A. O. , and G. Oboh . 2013. “Soybean Phenolic‐Rich Extracts Inhibit Key‐Enzymes Linked to Type 2 Diabetes (α‐Amylase and α‐Glucosidase) and Hypertension (Angiotensin I Converting Enzyme) In Vitro.” Experimental and Toxicologic Pathology 65, no. 3: 305–309. 22005499 10.1016/j.etp.2011.09.005

[fsn371884-bib-0002] Alsedfy, M. Y. , A. Ebnalwaled , M. Moustafa , A. A. Ebnalwaled , and A. H. Said . 2024. “Investigating the Binding Affinity, Molecular Dynamics, and ADMET Properties of Curcumin‐IONPs as a Mucoadhesive Bioavailable Oral Treatment for Iron Deficiency Anemia.” Scientific Reports 14, no. 1: 22027. 39322646 10.1038/s41598-024-72577-8 PMC11424638

[fsn371884-bib-0003] Andersen, J. M. , R. Barrangou , M. A. Hachem , et al. 2012. “Transcriptional Analysis of Prebiotic Uptake and Catabolism by *Lactobacillus acidophilus* NCFM.” 10.1371/journal.pone.0044409 PMC3446993 23028535

[fsn371884-bib-0004] Bagewadi, Z. , T. Yunus Khan , B. Gangadharappa , et al. 2023. “Molecular Dynamics and Simulation Analysis Against Superoxide Dismutase (SOD) Target of *Micrococcus luteus* With Secondary Metabolites From *Bacillus licheniformis* Recognized by Genome Mining Approach.” Saudi Journal of Biological Sciences 30: 103753. 37583871 10.1016/j.sjbs.2023.103753 PMC10424208

[fsn371884-bib-0005] Bakker, J. , and J. Bierau . 2012. “Purine and Pyrimidine Metabolism: Still More to Learn.” Nederlands Tijdschrift voor Klinische Chemie en Laboratoriumgeneeskunde 37, no. 1: 3–6.

[fsn371884-bib-0006] Bangar, S. P. , S. Suri , M. Trif , S. Punia Bangar , and F. Ozogul . 2022. “Organic Acids Production From Lactic Acid Bacteria: A Preservation Approach.” Food Bioscience 46: 101615.

[fsn371884-bib-0007] Benzie, I. F. , and J. J. Strain . 1996. “The Ferric Reducing Ability of Plasma (FRAP) as a Measure of ‘Antioxidant Power’: The FRAP Assay.” Analytical Biochemistry 239, no. 1: 70–76. 8660627 10.1006/abio.1996.0292

[fsn371884-bib-0008] Bifari, F. , C. Ruocco , I. Decimo , G. Fumagalli , A. Valerio , and E. Nisoli . 2017. “Amino Acid Supplements and Metabolic Health: A Potential Interplay Between Intestinal Microbiota and Systems Control.” Genes & Nutrition 12, no. 1: 27. 29043007 10.1186/s12263-017-0582-2 PMC5628494

[fsn371884-bib-0009] Bowers, K. J. , E. Chow , H. Xu , et al. 2006. “Scalable Algorithms for Molecular Dynamics Simulations on Commodity Clusters. Proceedings of the 2006 ACM/IEEE Conference on Supercomputing.”

[fsn371884-bib-0010] Brosnan, J. T. , and M. E. Brosnan . 2006. “The Sulfur‐Containing Amino Acids: An Overview.” Journal of Nutrition 136, no. 6: 1636S–1640S. 16702333 10.1093/jn/136.6.1636S

[fsn371884-bib-0011] Bülbül, M. , and S. Çokdinleyen . 2024. “Natural Lipase Inhibitors in Recent Years: A Review.” ChemistrySelect 9, no. 33: e202401091.

[fsn371884-bib-0012] C Borresen, E. , A. J Henderson , A. Kumar , et al. 2012. “Fermented Foods: Patented Approaches and Formulations for Nutritional Supplementation and Health Promotion.” Recent Patents on Food, Nutrition & Agriculture 4, no. 2: 134–140. 10.2174/2212798411204020134 PMC5175401 22702745

[fsn371884-bib-0013] Calsamiglia, S. , A. Ferret , and M. Devant . 2002. “Effects of pH and pH Fluctuations on Microbial Fermentation and Nutrient Flow From a Dual‐Flow Continuous Culture System.” Journal of Dairy Science 85, no. 3: 574–579. 11949862 10.3168/jds.S0022-0302(02)74111-8

[fsn371884-bib-0014] Chang, Y. , X.‐Q. Zhao , X. Zhang , and Y. Jiao . 2025. “Corn Steep Liquor as an Efficient Bioresource for Functional Components Production by Biotransformation Technology.” Food 14, no. 13: 2158. 10.3390/foods14132158 PMC12248704 40646908

[fsn371884-bib-0015] Chooi, Y. C. , C. Ding , and F. Magkos . 2019. “The Epidemiology of Obesity.” Metabolism 92: 6–10. 30253139 10.1016/j.metabol.2018.09.005

[fsn371884-bib-0016] Ciesielski, V. , P. Legrand , S. Blat , and V. Rioux . 2024. “New Insights on Pentadecanoic Acid With Special Focus on Its Controversial Essentiality: A Mini‐Review.” Biochimie 227: 123–129. 39395658 10.1016/j.biochi.2024.10.008

[fsn371884-bib-0017] Clarke, G. , K. N. Ting , C. Wiart , and J. Fry . 2013. “High Correlation of 2,2‐Diphenyl‐1‐Picrylhydrazyl (DPPH) Radical Scavenging, Ferric Reducing Activity Potential and Total Phenolics Content Indicates Redundancy in Use of All Three Assays to Screen for Antioxidant Activity of Extracts of Plants From the Malaysian Rainforest.” Antioxidants 2, no. 1: 1–10. 26787618 10.3390/antiox2010001 PMC4665400

[fsn371884-bib-0018] Cousin, E. , B. B. Duncan , C. Stein , et al. 2022. “Diabetes Mortality and Trends Before 25 Years of Age: An Analysis of the Global Burden of Disease Study 2019.” Lancet Diabetes & Endocrinology 10, no. 3: 177–192. 35143780 10.1016/S2213-8587(21)00349-1 PMC8860753

[fsn371884-bib-0019] Cui, L. , D.‐j. Li , and C.‐q. Liu . 2012. “Effect of Fermentation on the Nutritive Value of Maize.” International Journal of Food Science and Technology 47, no. 4: 755–760.

[fsn371884-bib-0020] D'Mello, J. F. 2003. Amino Acids in Animal Nutrition. 2nd ed. CABI Publishing.

[fsn371884-bib-0021] Egbujor, M. C. 2024. “Carboxylic Acids as Activators of NRF2: Antioxidant and Anti‐Inflammatory Effects.” Medicinal Chemistry 21: 1105–1126. 10.2174/0115734064335901241101025220 41607070

[fsn371884-bib-0022] Erukainure, O. L. , J. Nambooze , C. I. Chukwuma , A. Malloum , A. Aljoundi , and G. Elamin . 2024. “Computational and Theoretical Insights Into the Cytotoxic Prospects of Compounds Isolated From Elaeodendron Buchananii Against Leukemia.” Toxicology Reports 13: 101788. 39559566 10.1016/j.toxrep.2024.101788 PMC11570755

[fsn371884-bib-0023] Estoppey, A. , A. Vallat‐Michel , P. S. Chain , S. Bindschedler , and P. Junier . 2025. “Impact of Oxalic Acid Consumption and pH on the In Vitro Biological Control of Oxalogenic Phytopathogen Sclerotinia Sclerotiorum.” Journal of Fungi 11, no. 3: 191. 40137229 10.3390/jof11030191 PMC11942934

[fsn371884-bib-0024] Feng, L. , N. Tang , R. Liu , et al. 2021. “The Relationship Between Flavor Formation, Lipid Metabolism, and Microorganisms in Fermented Fish Products.” Food & Function 12, no. 13: 5685–5702. 34037049 10.1039/d1fo00692d

[fsn371884-bib-0025] Fraga‐Corral, M. , M. Carpena , P. Garcia‐Oliveira , A. G. Pereira , M. A. Prieto , and J. Simal‐Gandara . 2022. “Analytical Metabolomics and Applications in Health, Environmental and Food Science.” Critical Reviews in Analytical Chemistry 52, no. 4: 712–734. 33026841 10.1080/10408347.2020.1823811

[fsn371884-bib-0026] Hess, J. R. , and N. A. Greenberg . 2012. “The Role of Nucleotides in the Immune and Gastrointestinal Systems: Potential Clinical Applications.” Nutrition in Clinical Practice 27, no. 2: 281–294. 22392907 10.1177/0884533611434933

[fsn371884-bib-0027] Hijová, E. 2024. “Postbiotics as Metabolites and Their Biotherapeutic Potential.” International Journal of Molecular Sciences 25, no. 10: 5441. 38791478 10.3390/ijms25105441 PMC11121590

[fsn371884-bib-0028] Jabłońska‐Ryś, E. , A. Sławińska , K. Skrzypczak , and K. Goral . 2022. “Dynamics of Changes in pH and the Contents of Free Sugars, Organic Acids and LAB in Button Mushrooms During Controlled Lactic Fermentation.” Food 11, no. 11: 1553. 10.3390/foods11111553 PMC9180291 35681303

[fsn371884-bib-0029] Karigidi, K. O. , and C. O. Olaiya . 2020. “Antidiabetic Activity of Corn Steep Liquor Extract of Curculigo Pilosa and Its Solvent Fractions in Streptozotocin‐Induced Diabetic Rats.” Journal of Traditional and Complementary Medicine 10, no. 6: 555–564. 33134131 10.1016/j.jtcme.2019.06.005 PMC7588337

[fsn371884-bib-0030] Kim, Y. S. , Y. M. Lee , H. Kim , et al. 2010. “Anti‐Obesity Effect of Morus Bombycis Root Extract: Anti‐Lipase Activity and Lipolytic Effect.” Journal of Ethnopharmacology 130, no. 3: 621–624. 20669373 10.1016/j.jep.2010.05.053

[fsn371884-bib-0031] Krishnan, S. , N. Alden , and K. Lee . 2015. “Pathways and Functions of Gut Microbiota Metabolism Impacting Host Physiology.” Current Opinion in Biotechnology 36: 137–145. 26340103 10.1016/j.copbio.2015.08.015 PMC4688195

[fsn371884-bib-0032] Kumar, M. , M. K. Tripathi , and P. Kaur . 2024. “Molecular Dynamics and Its Significance in Drug Discovery.” In Structure‐Based Drug Design, 149–175. Springer.

[fsn371884-bib-0033] Lee, T.‐W. , Y.‐B. Song , C. Y. Kim , J. H. Lee , and B. H. Lee . 2025. “Regulation of Glucose Uptake Level by Black Corn‐Derived Anthocyanins at the Small Intestinal α‐Glucosidases and Different Types of Glucose Transporters.” Journal of Agricultural and Food Chemistry 73: 14523–14532. 40439575 10.1021/acs.jafc.5c04779

[fsn371884-bib-0034] Liu, H. , B. Su , M. Guo , and J. Wang . 2024. “Exploring R&D Network Resilience Under Risk Propagation: An Organizational Learning Perspective.” International Journal of Production Economics 273: 109266.

[fsn371884-bib-0035] Liu, W. , X. Cui , Y. Zhong , R. Ma , B. Liu , and Y. Xia . 2023. “Phenolic Metabolites as Therapeutic in Inflammation and Neoplasms: Molecular Pathways Explaining Their Efficacy.” Pharmacological Research 193: 106812. 37271425 10.1016/j.phrs.2023.106812

[fsn371884-bib-0036] López‐Garzón, C. S. , and A. J. Straathof . 2014. “Recovery of Carboxylic Acids Produced by Fermentation.” Biotechnology Advances 32, no. 5: 873–904. 24751382 10.1016/j.biotechadv.2014.04.002

[fsn371884-bib-0037] Luise, D. , T. Chalvon‐Demersay , F. Correa , et al. 2023. “A Systematic Review of the Effects of Functional Amino Acids on Small Intestine Barrier Function and Immunity in Piglets.” Animal 17: 100771. 37003917 10.1016/j.animal.2023.100771

[fsn371884-bib-0038] Marco, M. L. , D. Heeney , S. Binda , et al. 2017. “Health Benefits of Fermented Foods: Microbiota and Beyond.” Current Opinion in Biotechnology 44: 94–102. 27998788 10.1016/j.copbio.2016.11.010

[fsn371884-bib-0039] Martínez, L. 2015. “Automatic Identification of Mobile and Rigid Substructures in Molecular Dynamics Simulations and Fractional Structural Fluctuation Analysis.” PLoS One 10, no. 3: e0119264. 25816325 10.1371/journal.pone.0119264 PMC4376797

[fsn371884-bib-0040] Martyna, G. J. , D. J. Tobias , and M. L. Klein . 1994. “Constant Pressure Molecular Dynamics Algorithms.” Journal of Chemical Physics 101, no. 4177: 1063.

[fsn371884-bib-0041] Mira, N. P. , R. Marshall , M. J. F. Pinheiro , et al. 2024. “On the Potential Role of Naturally Occurring Carboxylic Organic Acids as Anti‐Infective Agents: Opportunities and Challenges.” International Journal of Infectious Diseases 140: 119–123. 38325748 10.1016/j.ijid.2024.01.011

[fsn371884-bib-0042] Mohamed, A. I. , O. L. Erukainure , H. Ismail , and M. S. Islam . 2025. “Exploring the Chemical Profile, Antioxidants, and Anti‐Diabetic Properties of Coffee Beans From Selected East African Countries: A Comparative In Vitro and Computational Study.” Food Science & Nutrition 13, no. 7: e70527. 40666827 10.1002/fsn3.70527 PMC12259390

[fsn371884-bib-0043] Nagarajan, M. , B. Rajasekaran , and K. Venkatachalam . 2022. “Microbial Metabolites in Fermented Food Products and Their Potential Benefits.” International Food Research Journal 29, no. 3: 466–486.

[fsn371884-bib-0044] Okeke, C. A. , C. N. Ezekiel , M. Sulyok , et al. 2018. “Traditional Processing Impacts Mycotoxin Levels and Nutritional Value of Ogi–a Maize‐Based Complementary Food.” Food Control 86: 224–233.

[fsn371884-bib-0045] Ozoh, C. , C. Imoisi , and J. Iyasele . 2023. “Effect of pH and Duration of Fermentation on the Quality Characteristics of Garri.” Pakistan Journal of Nutrition 22: 45–51.

[fsn371884-bib-0046] Pang, Z. , Y. Lu , G. Zhou , et al. 2024. “MetaboAnalyst 6.0: Towards a Unified Platform for Metabolomics Data Processing, Analysis and Interpretation.” Nucleic Acids Research 52, no. W1: W398–W406. 38587201 10.1093/nar/gkae253 PMC11223798

[fsn371884-bib-0047] Pfeuffer, M. , and A. Jaudszus . 2016. “Pentadecanoic and Heptadecanoic Acids: Multifaceted Odd‐Chain Fatty Acids.” Advances in Nutrition 7, no. 4: 730–734. 27422507 10.3945/an.115.011387 PMC4942867

[fsn371884-bib-0048] Pobereżny, J. , E. Wszelaczyńska , R. Lamparski , et al. 2023. “The Impact of Spring Wheat Species and Sowing Density on Soil Biochemical Properties, Content of Secondary Plant Metabolites and the Presence of Oulema Ssp.” PeerJ 11: e14916. 36860764 10.7717/peerj.14916 PMC9969853

[fsn371884-bib-0049] Sethi, J. K. , and G. S. Hotamisligil . 2021. “Metabolic Messengers: Tumour Necrosis Factor.” Nature Metabolism 3, no. 10: 1302–1312. 10.1038/s42255-021-00470-z 34650277

[fsn371884-bib-0050] Shai, L. J. , P. Masoko , M. P. Mokgotho , et al. 2010. “Yeast Alpha Glucosidase Inhibitory and Antioxidant Activities of Six Medicinal Plants Collected in Phalaborwa, South Africa.” South African Journal of Botany 76, no. 3: 465–470.

[fsn371884-bib-0051] Sharma, R. , B. Diwan , B. P. Singh , and S. Kulshrestha . 2022. “Probiotic Fermentation of Polyphenols: Potential Sources of Novel Functional Foods.” Food Production, Processing and Nutrition 4, no. 1: 21.

[fsn371884-bib-0052] Sharma, R. , P. Garg , P. Kumar , S. K. Bhatia , and S. Kulshrestha . 2020. “Microbial Fermentation and Its Role in Quality Improvement of Fermented Foods.” Fermentation 6, no. 4: 106.

[fsn371884-bib-0053] Sim, L. , K. Jayakanthan , S. Mohan , et al. 2010. “New Glucosidase Inhibitors From an Ayurvedic Herbal Treatment for Type 2 Diabetes: Structures and Inhibition of Human Intestinal Maltase‐Glucoamylase With Compounds From Salacia Reticulata.” Biochemistry 49, no. 3: 443–451. 20039683 10.1021/bi9016457

[fsn371884-bib-0054] Singh, G. , S. Suresh , V. K. Bayineni , et al. 2015. “Lipase Inhibitors From Plants and Their Medical Applications.” International Journal of Pharmacy and Pharmaceutical Sciences 7, no. 1: 1–5.

[fsn371884-bib-0055] Susilowati, A. , P. Lotulung , and Y. Maryati . 2019. “Difference in Characteristic of Concentrate Powder of Corn ( *Zea mays var. indentata* ) Fermented by Bifidobacterium brevis as Natural Folic Acid Fortificant.” In Journal of Physics: Conference Series. IOP Publishing.

[fsn371884-bib-0056] Tabatabaei‐Malazy, O. , B. Larijani , and M. Abdollahi . 2015. “Targeting Metabolic Disorders by Natural Products.” Journal of Diabetes and Metabolic Disorders 14, no. 1: 57. 26157708 10.1186/s40200-015-0184-8 PMC4495701

[fsn371884-bib-0057] Tamang, J. P. , K. Watanabe , and W. H. Holzapfel . 2016. “Diversity of Microorganisms in Global Fermented Foods and Beverages.” Frontiers in Microbiology 7: 377. 27047484 10.3389/fmicb.2016.00377 PMC4805592

[fsn371884-bib-0058] Taverniti, V. , and S. Guglielmetti . 2011. “The Immunomodulatory Properties of Probiotic Microorganisms Beyond Their Viability (Ghost Probiotics: Proposal of Paraprobiotic Concept).” Genes & Nutrition 6, no. 3: 261–274. 21499799 10.1007/s12263-011-0218-x PMC3145061

[fsn371884-bib-0059] Thorakkattu, P. , A. C. Khanashyam , K. Shah , et al. 2022. “Postbiotics: Current Trends in Food and Pharmaceutical Industry.” Food 11, no. 19: 3094. 10.3390/foods11193094 PMC9564201 36230169

[fsn371884-bib-0060] Thuy, C. X. , V. T. Pham , T. T. N. H. Nguyen , et al. 2024. “Effect of Fermentation Conditions (Dilution Ratio, Medium Ph, Total Soluble Solids, and *Saccharomyces cerevisiae* Yeast Ratio) on the Ability to Ferment Cider From Tamarillo ( *Solanum betaceum* ) Fruit.” Journal of Food Processing & Preservation 2024, no. 1: 8841207.

[fsn371884-bib-0061] Toh, J. Z. K. , X.‐H. Pan , P. W. L. Tay , et al. 2022. “A Meta‐Analysis on the Global Prevalence, Risk Factors and Screening of Coronary Heart Disease in Nonalcoholic Fatty Liver Disease.” Clinical Gastroenterology and Hepatology 20, no. 11: 2462–2473.e10. 34560278 10.1016/j.cgh.2021.09.021

[fsn371884-bib-0062] Tonolo, F. , A. Folda , S. Ferro , et al. 2023. “Fermentation of Corn By‐Products: From Agrifood Waste to Higher Value Antioxidant Products.” Fermentation 9, no. 4: 373.

[fsn371884-bib-0063] Tundis, R. , M. R. Loizzo , and F. Menichini . 2010. “Natural Products as α‐Amylase and α‐Glucosidase Inhibitors and Their Hypoglycaemic Potential in the Treatment of Diabetes: An Update.” Mini‐Reviews in Medicinal Chemistry 10, no. 4: 315–331. 20470247 10.2174/138955710791331007

[fsn371884-bib-0064] Utpott, M. , E. Rodrigues , A. de Oliveira Rios , et al. 2022. “Metabolomics: An Analytical Technique for Food Processing Evaluation.” Food Chemistry 366: 130685. 34333182 10.1016/j.foodchem.2021.130685

[fsn371884-bib-0065] van Hylckama Vlieg, J. E. , P. Veiga , C. Zhang , et al. 2011. “Impact of Microbial Transformation of Food on Health—From Fermented Foods to Fermentation in the Gastro‐Intestinal Tract.” Current Opinion in Biotechnology 22, no. 2: 211–219. 21247750 10.1016/j.copbio.2010.12.004

[fsn371884-bib-0066] Wang, Y. , G. Corrieu , and C. Béal . 2005. “Fermentation pH and Temperature Influence the Cryotolerance of *Lactobacillus acidophilus* RD758.” Journal of Dairy Science 88, no. 1: 21–29. 15591363 10.3168/jds.S0022-0302(05)72658-8

[fsn371884-bib-0067] Wegh, C. A. , S. Y. Geerlings , J. Knol , C. A. M. Wegh , G. Roeselers , and C. Belzer . 2019. “Postbiotics and Their Potential Applications in Early Life Nutrition and Beyond.” International Journal of Molecular Sciences 20, no. 19: 4673. 31547172 10.3390/ijms20194673 PMC6801921

[fsn371884-bib-0068] Xie, F. , Z. Liu , M. Liu , L. Chen , W. Ding , and H. Zhang . 2020. “Amino Acids Regulate Glycolipid Metabolism and Alter Intestinal Microbial Composition.” Current Protein & Peptide Science 21, no. 8: 761–765. 32072901 10.2174/1389203721666200219100216

[fsn371884-bib-0069] Yang, F. , C. Chen , D. Ni , et al. 2023. “Effects of Fermentation on Bioactivity and the Composition of Polyphenols Contained in Polyphenol‐Rich Foods: A Review.” Food 12, no. 17: 3315. 10.3390/foods12173315 PMC10486714 37685247

[fsn371884-bib-0070] Zhou, K. , J. Yu , Y. Ma , et al. 2022. “Corn Steep Liquor: Green Biological Resources for Bioindustry.” Applied Biochemistry and Biotechnology 194, no. 7: 3280–3295. 35349086 10.1007/s12010-022-03904-w

[fsn371884-bib-0071] Zúñiga, M. , V. Monedero , and M. J. Yebra . 2018. “Utilization of Host‐Derived Glycans by Intestinal Lactobacillus and Bifidobacterium Species.” Frontiers in Microbiology 9: 1917. 30177920 10.3389/fmicb.2018.01917 PMC6109692

